# Recent Treatment Advances and Novel Therapies in Pancreas Cancer: A Review

**DOI:** 10.1007/s12029-013-9561-z

**Published:** 2013-12-17

**Authors:** Matias E. Valsecchi, Enrique Díaz-Cantón, Máximo de la Vega, Susan J. Littman

**Affiliations:** 1 Department of Medical Oncology, Thomas Jefferson University, Philadelphia, PA 19107 USA; 2 Centro de Educación Médica e Investigaciones Clínicas (CEMIC), Buenos Aires, Argentina

**Keywords:** Pancreatic cancer, FOLFIRINOX, Nab-paclitaxel, Gemcitabine, Erlotinib, Gastrointestinal cancer

## Abstract

**Purpose:**

Over the last couple of years, we have witnessed the availability of a wide variety of different therapeutic agents and the identification of effective combinations of existing ones that have transformed the way we approach and treat pancreatic cancer. Proof of this are the recent validations that combinations of conventional chemotherapy drugs, the FOLFIRINOX regimen and gemcitabine plus nab-paclitaxel, significantly improves clinical outcomes in patients with metastatic disease. However, deeper and more sophisticated understanding of the biology of this cancer as well as the ability to develop better and perhaps more precise drugs predict that the landscape may be changing even more.

**Methodology and Results:**

In this review, we will summarize the most recent treatment advances including FOLFIRINOX, gemcitabine plus nab-paclitaxel and discuss novel approaches such as immune-mediated therapies, drugs that disrupt the tumor-stromal compartment, PARP inhibitors for BRCA pathway-deficient pancreatic cancer and new generations of conventional chemotherapeutics, which are in early phases of clinical development and have shown promising early results. We will also discuss some examples of drugs that failed, despite very good preliminary data, in order to appraise the lessons learned from these negative clinical trials. Lastly, we will comment on ongoing adjuvant and neoadjuvant trials.

**Conclusion:**

We hope that at least some of these will result in positive trials and add to our armamentarium for treating this challenging malignancy.

## Introduction

Pancreatic ductal adenocarcinoma (PDA) is undoubtedly one of the most lethal cancers. Survival rates are still poor despite the significant efforts over the years have been devoted to studying this disease. Since the mid 1990s, gemcitabine emerged as the main therapeutic armament to fight this devastating disease; however, its real clinical benefit is a modest 1-month extension in the overall survival (OS; 5.6 vs. 4.4 months) when compared to 5-FU [1, 2]. Nevertheless, these findings were encouraging and the next decade of research involved numerous studies aimed at improving gemcitabine efficacy and testing gemcitabine doublets, resulting in minor improvements in OS.

Although pessimism has reigned for some time regarding the likelihood of achieving substantial clinical improvement in outcomes in PDA, the last few years have witnessed the emergence of a variety of therapeutic strategies that have already changed our daily practice. The first was the verification that a combination of chemotherapy agents (FOLFIRINOX) could improve all the relevant clinical outcomes in patients with metastatic disease [3]. The confluence of a deeper and more sophisticated understanding of pancreatic tumor biology plus the development of better multi-agent combinations we suspect will foretell the availability of additional effective therapies in the near future.

The main objective of this review is to summarize recent therapeutic advances that represent a substantial change in the way we approach PDA in 2013. Pursuing this objective, we will describe the new multi-agent combinations recently FDA-approved, as well as drugs that are in the earlier phases of development and that have shown promising results. Lastly, we will also discuss some examples of approaches that failed, despite good preliminary data, to evaluate the lessons learned from these negative clinical trials.

## Recent Treatment Advances Already Available in Clinical Practice

From a clinical perspective, PDA is divided into three main categories: surgically resectable, locally advanced (LA), and metastatic. We often say that surgery offers the only chance of cure in PDA. While this statement is true, the actual cure rate is low—about 3–5 % at 5 years [4]. Thus, advances in the medical treatment are essential to obtain improvements in OS even with early disease.

Three multi-agent chemotherapy regimens have resulted in positive findings: FOLFIRINOX, gemcitabine + nab-paclitaxel, and gemcitabine + erlotinib (Table 1).

**Table 1:** Summary of positive clinical trials that lead to practice changes in metastatic PDA

Intervention	Study description	No. of patients	Comparison	Median OS	Median DFS or TTP	ORR	1-year survival	References
*Gemcitabine*								
Phase 3	Randomized, multicenter international	160	Gemcitabine vs. 5-Flourouracil	5.65 vs. 4.41 months	9 vs. 4 weeks	5.4 % vs. 0 %	18 % vs. 2 %	Burris H, et al. [1]
*FOLFIRINOX*								
Phase 2	Single arm; multicenter	47	None	10.2 (8.1–14.4 months)	8.2 (5.3–11.6 months)	26 % (13–39 %)	43 %	Conroy et al. [5]
Phase 3	Phases 2–3, randomized, placebo-controlled, multicenter, international.	342	FOLFIRINOX vs. Gemcitabine	11.1 vs. 6.8 months	6.4 vs. 3.3 months	31.6 % vs. 9.4 %	48 % vs. 20 %	Conroy et al. [3]
*Nab*-*Paclitaxel*								
Phase 2	Single arm, open-label, multicenter, phase I/II	67	None	12.2 (8.9–18 months)	7.9 (5.8–11 months)	46 % (N/A)	48 %	Von Hoff et al. [21]
Phase 3	Randomized phase III, multicenter	861	Gemcitabine ± Nab-Paclitaxel or Placebo	8.5 vs. 6.7 months	5.5 vs. 3.7 months	23 % vs. 7 %	35 % vs. 22 %	Von Hoff et al. [22]
*Erlotinib*								
Phase 3	Randomized, double-blinded, placebo-controlled, international	569	Gemcitabine ± erlotinib or Placebo	6.24 vs. 5.91 months	3.75 vs. 3.55 months	8.6 % vs. 8.0 %	23 % vs. 17 %	Moore M, et al. [25]

### FOLFIRINOX

FOLFIRINOX, a regimen composed of four drugs (Folinic acid 400 mg/m^2^, 5-FU bolus 400 mg/m^2^ followed by 48 h infusional 5-FU 2,400 mg/m^2^, Irinotecan 180 mg/m^2^, and oxaliplatin 85 mg/m^2^) is currently considered a first-line treatment option for metastatic PDA. Prior to the testing of this multi-agent regimen, several trials explored the addition of a second agent to gemcitabine in metastatic disease. Gemcitabine was combined with platinum drugs (pooled analysis, HR = 0.85, *P* = 0.01), with fluoropyrimidine (pooled analysis, HR = 0.90, *P* = 0.03) as well as topoisomerase inhibitors (pooled analysis, HR = 0.99, *P* = 0.80) [2]. The median OS ranged from 6 to 9 months, and the overall effect of adding a second drug was a modest but detectable 10 % (CI: 3–15 %) improvement in OS. This slight improvement in efficacy of gemcitabine doublets was the rationale used by French investigators to study a multi-drug regimen that did not include gemcitabine. They initially conducted a robust Phase II trial demonstrating an impressive 26 % response rate and a median OS of 10.2 months [5]. Based on this and the fact that toxicity although significant was manageable, investigators of the PRODIGE Intergroup designed a randomized Phases II–III clinical trial comparing FOLFIRINOX to single-agent gemcitabine as first-line treatment for metastatic PDA [3]. A number of 342 patients with histologically confirmed metastatic disease and no significant comorbidities were enrolled. The primary endpoint was OS; secondary endpoints were PFS, tumor response (RECIST), safety, and quality of life. The study was closed early when an interim analysis showed a striking 4.3 months prolongation in median OS favoring FOLFIRINOX (11.1 vs. 6.8 months; HR = 0.57, *P* < 0.001). Median PFS was 6.4 vs. 3.3 months (*P* < 0.001) and ORR was 31 % vs. 9.5 % (*P* < 0.001). There was also a significant increase in grades 3–4 toxicities mainly related to cytopenias, neutropenic fever, diarrhea, vomiting, and peripheral neuropathy (all with *P* < 0.01).

Since the publication of this pivotal clinical trial, many single and multi-institutional studies have been reported confirming the efficacy of this regimen. Two main issues have arisen regarding the general application of these findings. The first pertains to the ability to utilize the entry criteria of the Accord II trial for non-study metastatic PDA patients. To address this, investigators from British Columbia performed a retrospective review of 100 consecutive cases in their database to determine the proportion of “real-world” patients that would have met eligibility. They found that only 26 patients would have been eligible, with the main reasons for exclusion being older age (22 %), poor performance status (64 %), and organ dysfunction (28 %) [6]. The second issue relates to the tolerability of this regimen outside of the highly controlled environment of a clinical trial. “Tolerability” of FOLFIRINOX remains controversial with some reports showing easily manageable side effects [7, 8], while others demonstrate significant toxicity with 32 % hospitalization [9] and treatment discontinuation in one third of the patients [10]. Conscious of these limitations, Mahaseth and colleagues proposed a modified FOLFIRINOX in which the 5-FU bolus was removed and growth factor prophylaxis was used routinely. Authors reported significantly less grade 3–4 toxicity but similar activity [11]. Additionally, in a small retrospective analysis of 35 patients treated at the Yale Cancer Center, 29 (85 %) who received dose-attenuated FOLFIRINOX showed no significant reduction in response rate [12]. These results are reassuring and have enhanced the acceptability of this regimen among general oncologists who frequently adjust drug doses based on the individual patient's performance status. Of concern, however, is the possibility that personal physician modifications effect survival outcomes. This illustrates what is common knowledge today that a minority of metastatic patients are really candidates for FOLFIRINOX. We certainly need better biomarkers to assist us in determining which patients really benefit from this regimen [13].

FOLFIRINOX has also been investigated in earlier stages of disease. Kharofa and colleagues retrospectively evaluated 12 patients with unresectable PDA and who received neo-adjuvant FOLFIRINOX followed by concurrent chemo-radiation therapy with either gemcitabine or 5-FU. Seven patients (58 %) had R0 resection and the median survival was not reached after 13 months of follow-up [14]. In a similar retrospective analysis of 18 patients with unresectable and borderline-resectable PDA, seven (39 %) were taken to the OR after neoadjuvant FOLFIRINOX, five having R0, one R1 resections and one was not resectable [15]. Toxicity in this population was significant with grades 3–4 toxicities including neutropenia (22 %), neutropenic fever (17 %), thrombocytopenia (11 %), fatigue (11 %), and diarrhea (11 %). In a second retrospective single-institution experience with FOLFIRINOX followed by chemo-radiation in LA PDA ORR was 27.3 % and median PFS was 11.7 %. While 22 % (5 of 22) were able to undergo R0 resections, the durability of these responses were short with three developing distant metastases after 5 months [9]. Lastly, investigators from the University of Pittsburgh reported that 7 out of 25 patients with unresectable or borderline resectable PDA underwent surgical resection after receiving FOLFIRINOX [16].

### Nab-Paclitaxel

Nab-paclitaxel (Abraxane®) is an albumin-bound, 130-nM particle, formulation of paclitaxel administered as a colloidal suspension [17]. This drug was originally developed to overcome the well-known anaphylactic reactions associated with the conventional Cremophor EL formulation of paclitaxel. However, this change in formulation presumably related to the intra-tumoral drug delivery resulted in efficacy improvement in PDA [18]. The molecular mechanism of this therapy enhancement is not fully understood. It was originally thought to be related to high expression of the protein SPARC, which is known to bind albumin, by the tumoral stromal cells. However, recent investigations suggested that an albumin receptor (gp60) on endothelial cells could be involved in transporting paclitaxel into the tumoral interstitial space [19]. Preclinical studies showed that the addition of nab-paclitaxel to gemcitabine increases intratumoral gemcitabine levels due to a marked decrease in the primary gemcitabine metabolizing enzyme, cytidine deaminase, suggesting synergy between these two drugs may contribute to clinical benefit in pancreatic cancer [20].

The first proof of principle that nab-paclitaxel was effective in metastatic PDA came from a Phase I/II trial in which nab-paclitaxel plus gemcitabine was tested in 67 metastatic patients. After reaching a MTD of 125 mg/m^2^, 44 patients completed the Phase II portion of the study showing a median PFS of 7.9 months (95 % CI: 6–11 months), a median OS of 12.2 months (95 % CI: 9–18 months), and a 1-year survival of 48 % [21].

The open label, international, randomized Phase III trial (MPACT study) was initially presented in 2013 at ASCO GI and supports the use of the nab-paclitaxel/gemcitabine combination in metastatic PDA. Investigators randomized 861 patients to either gemcitabine 1,000 mg/m^2^ alone for 7 weeks followed by a rest week vs. nab-paclitaxel 125 mg/m^2^ plus gemcitabine 1,000 mg/m^2^ for 3 weeks followed by a rest week and treated until progression. The primary endpoint was OS and secondary endpoints included PFS, ORR, safety, and tolerability of this combination. The median OS was 8.5 months for the combination vs. 6.7 months for gemcitabine alone (HR = 0.72; *P* < 0.001). Moreover, the time to treatment failure was significantly prolonged with the combination from 3.5 to 5.1 months (HR = 0.70; *P* < 0.001). 1-year survival was also improved (35 % vs. 22 %, *P* < 0.001) [22]. Sub-analysis, presented at ASCO 2013, showed liver metastases and poor PS (KPS < 80) predicted the greatest advantage of the combination. Based on this data, the FDA has approved nab-paclitaxel plus gemcitabine as a first-line treatment option for patients with metastatic PDA citing a high unmet need and few effective treatments (announced in September 6, 2013). As in the case of FOLFIRINOX, nab-paclitaxel is being tested in earlier stages of disease [23, 24].

### Erlotinib

Erlotinib plus gemcitabine is the only other drug combination that has proven to be of some benefit in a randomized Phase III clinical trial for patients with LA and metastatic PDA. In 2007, Canadian investigators presented the results of the NCI PA.3 study in which 569 patients were randomly assigned to gemcitabine 1,000 mg/m^2^ weekly for 7 weeks followed by a week rest followed by 4-week cycles plus erlotinib 100–150 mg or placebo [25]. Patients were treated until disease progression or toxicity. The results showed a minimal improvement in median OS (6.2 vs. 5.9 months; HR = 0.82, *P* = 0.038) and an absolute 6 % benefit in 1-year OS (23 % vs. 17 %, *P* = 0.023). The study also showed a statistically significant improvement in DFS but no difference in ORR. Patients receiving erlotinib experienced higher frequencies of rash, diarrhea, infection, and stomatitis. There were six protocol-related deaths, all in the arm containing erlotinib attributed to interstitial pneumonitis (2), sepsis (2), neutropenic sepsis (1), and CVA (1). A total of eight patients, seven in the erlotinib group, developed an interstitial lung disease-like syndrome. Since the publication of this clinical trial, there has been significant controversy as to whether the short duration of benefit is balanced by the risks. However, given the lack of effective treatments, the combination did achieve FDA approval in November 2005.

Due to their greater potential for benefit, FOLFIRINOX and gemcitabine plus nab-paclitaxel combination chemotherapy are the preferable options for patients with better performance status and organ function. Notwithstanding, and for reasons not entirely clear, some patients respond unusually well to erlotinib plus gemcitabine. A remarkable observation reported in NCI PA.3 is the association between the development of cutaneous rash and clinical outcome in erlotinib-treated patients. Patients with grade ≥ 2 skin rash had a 43 % 1-year survival and 10.5 months median OS in comparison 16 % and 5.3 months for those without rash, respectively (*P* < 0.001). Although technically a negative trial, with overall outcomes nearly identical to NCI PA.3, a similar correlation regarding the predictive value of the skin rash was made in an open label Phase II study of erlotinib 100 mg daily and fixed-dose rate gemcitabine 1,000 mg/m^2^ [26]. Patients with grade ≥ 2 rash had a significantly higher chance of treatment benefit (OS 42 vs. 15 weeks, *P* = 0.03). The striking similarity in survival between this trial and the NCI PA.3 also suggests that the pharmacokinetic modulation of gemcitabine appears to be of little clinical advantage [27]. In the Phase II RACHEL trial, erlotinib was dose escalated from 150 to 250 mg/day until at least grade 2 skin rash was obtained. Disappointingly, dose escalation did not result in clinical benefit [28].

## Novel Therapies in Early Phases of Clinical Development

There are now numerous follow-on trials testing FOLFIRINOX and gemcitabine-nab-paclitaxel in earlier disease stages. Most notably, it is clear that both are effective in LA PDA and the value of radiation in addition to multi-agent chemotherapy is still to be determined. While we wait for these trials to mature it is interesting to also speculate about a number of novel therapies in the pipeline. Caution must prevail when considering these therapies, as encouraging results in early phases of development do not necessarily translate into clinically relevant outcomes, when tested in larger populations or when compared with the standard of care.

### Immunotherapy

We have recently witnessed a strong resurgence for immune therapies in cancer treatments. The approval of sipuleucel-T immunotherapy and ipilimumab to treat prostate and melanoma cancers are just two examples [29, 30]. PDA does not seem to be the exception, although due to the aggressive biology of this malignancy immune-based therapies have generally been combined with chemotherapy.

A comprehensive review of the potential tumor-specific antigens as well as the strategies used against PDA can be found on other reviews and is beyond the scope of this overview [31]. Briefly, potential methods of immune activation against pancreatic tumors include: (a) whole-cell vaccines (e.g., genetically modified to over-express a particular epitope); (b) peptide vaccines designed to boost CD 8+ cytotoxic T-lymphocyte response; and (c) DNA vaccines in which modified DNA coding for a target antigen is inserted into a vector which is directly taken up into tumoral cells.

Algenpantucel-L immunotherapy, a whole-cell allogenic pancreatic cancer vaccine developed by NewLink Genetics (Ames, IA, USA) is based on the concept of hyperacute rejection that occurs with xenotransplantation. The vaccine is composed of two irradiated human pancreatic cell lines that have been genetically modified to over-express murine α(1,3)-galactosyltransferase resulting in expression of α-galactosyl (α-gal) epitopes on membrane glycoproteins and glycolipids. Since these epitopes are not expressed by human cells an immediate hyperacute rejection response occurs similar to that observed with transplant rejection. Vaccine exposure results in antibody-dependent cell-mediated cytotoxicity with lysis of cells containing the α-gal epitopes and the consequent development of a strong T-cell mediated anti-tumor immunity. A multi-institutional, open-label, Phase 2 trial was published [32]. A number of 73 patients with resected PDA (R0 or R1) received algenpantucel-L in addition to conventional gemcitabine followed by 5-FU based chemo-radiation, as adjuvant treatment. Primary endpoint was 12-month DFS; secondary endpoints included OS and toxicity. 80 % patients had positive lymph nodes. No significant side effects were reported other than fatigue and local skin reactions. The median DFS was 14 months and the 1-year DFS was 62 %. Median OS was not reached but the 1-year OS was 86 %. Higher vaccine doses resulted in improved DFS (80 % vs. 51 %; *P* = 0.02) and OS (96 % vs. 79 %; *P* = 0.053) at 12 months. A Phase III clinical trial with similar design and a target of 722 subjects has just completed enrollment.

Other Phase II clinical trials were reported with less encouraging results. Lutz et al. [33] designed a single-institution adjuvant study of 60 patients with resected PDA who received an initial injection of an irradiated GM-CSF transfected whole cell vaccine followed by 5-FU-based chemo-radiation therapy and up to four additional immunizations. Median DFS and OS were 17.3 and 24.8 months, respectively. Overall, the vaccine was well tolerated. A randomized, multicenter, placebo-controlled study with G17DT (Gastrimmune), an anti-gastrin17 immunogen, showed a non-significant improvement in OS compared to placebo in advanced disease [34]. Yanagimoto and colleagues [35] reported a median survival of 9 months with a 1-year survival rate of 38 % in 21 patients with metastatic disease who received a personalized peptide vaccine to HLA in combination with standard weekly gemcitabine.

### Stromal Disruption

Tumor stroma was once considered an inert bystander but is now regarded as a key component of tumor biology. The stroma of PDA is composed of an abundant extra-cellular matrix that does not act simply as a mechanical support but actively facilitates tumor development [36, 37]. The complex interplay between cancer cells and the various components of the tumor microenvironment has led to the testing of novel therapies to interrupt these biological processes.

The first mechanism explored was the use of inhibitors of the matrix metalloproteinases. Unfortunately, multiple studies showed that this approach is quite ineffective [38]. A second strategy involves the inhibition of cell-stroma interactions fueled by soluble factors secreted from stromal cells and used by tumors to enable the development of tumor vasculature or tumor growth. Bevacizumab (Genentech/Roche) showed no activity in multiple Phase I/II trials [39]. Sorafenib was also ineffective in a Phase II study [40]. Dovitinib (an inhibitor of FGFR-3) is being tested in combination with gemcitabine/capecitabine in a Phase I trial. A randomized Phase II study of tivantinib (small molecule inhibitor of c-Met) vs. gemcitabine in treatment-naive patients with advanced PDA was completed and results are pending. In a French Phase II trial, 22 patients with LA or metastatic PDA received oral masitinib (inhibitor of c-kit and PDGFR kinases) combined with standard gemcitabine. The reported median TTP and OS were 6.4 months and 7.1 months, respectively [41]. A Phase III study recently updated in unresectable PDA using gemcitabine plus masitinib vs. placebo showed no benefit in OS (7.7 vs. 7.0 months); however, patients with a deleterious genomic biomarker had a significant improvement in OS (11 vs. 5 months, *P* < 0.001) [42]. Rigosertib, a phosphatidylinositol-3-kinase inhibitor, showed activity in a Phase I trial [43] and is currently being tested in a Phase II/III randomized clinical trial. Trabedersen, an antisense molecule which is specifically designed to target TGF-β2 mRNA, showed encouraging results in Phase I/II and a Phase II is planned [44].

Three developmental pathways (WNT, notch, and the hedgehog) have been implicated in induction of epithelial-to-mesenchymal transformation as well as pancreatic cancer progression and maintenance of cancer stem cells [45]. Many new compounds targeting these pathways are being tested. A recent Phase II clinical trial with hedgehog pathway inhibitor saridegib (Infinity Pharmaceuticals) showed no benefit when added to gemcitabine; but some activity was seen in a Phase Ib trial with FOLFIRINOX [46]. Several Phase II trials of hedgehog pathway inhibitor vismodegib (Genentech/Curtis) and Phase I trials of LDE225 (Novartis) in combination with gemcitabine are ongoing. Notch inhibitors, RO4929097 and MK0752, concluded Phase I studies and will move forward into Phase II [47, 48]. We will have to wait until all these studies are completed before any formal conclusion can be made about this group of drugs.

### PARP Inhibitors

Studies have demonstrated an increased risk of PDA among relatives of patients with PDA 7.8 % compared to 0.6 % in controls–even after adjusting for other factors [49]. In Ashkenazi Jews, a family history of PDA is a strong predictor for BRCA2 mutation (OR = 6.1) [50]. Familial pancreatic cancer represents approximately 5 % of all newly diagnosed PDA Genetic predisposition to PDA is polygenic; seen individuals with mutations in Fanconi anemia pathway genes (BRCA2, BRCA1, and PALB2), DNA mismatch repair genes (MSH2, MSH6, MLH1, and PMS2), CDK2A/p16 (FAMMM syndrome), PRSS1 (hereditary pancreatitis), and LKB1/STK11 (Puetz-Jeghers) [51].

BRCA1/2 harbors particular therapeutic interest because homozygous mutant pancreatic cells are exquisitely sensitive to DNA cross-linking agents such as mitomycin C and cisplatin as well as PARP (Poly[ADP-ribose] polymerase) inhibitors [52]. There are several PARP-1 inhibitors in clinical trials. At ASCO GI 2103, Pishvaian and colleagues presented preliminary results of the Phase I portion of their trial combining veleparib (ABT-888, Abbott Laboratories) with FOLFOX in metastatic PDA. Of 22 patients treated on the Phase I portion of the study, the two best responders were patients with BRCA2 mutations; one had a PR with stable disease after 17 months and the second had a CR with normalization of CA 19–9 after 10 months [53]. The Phase II portion of the study was modified to include patients with known or suspected BRCA genetic mutations. Another Phase II clinical trial of patients with confirmed BRCA1/2 mutation treated with olaparib (AZD2281, AstraZeneca) monotherapy was presented at ASCO 2013. A number of 23 patients were included resulting in 1 CR, 4 PRs, and 8 SD for an ORR of 21.7, and 40.9 % 1-year survival [54]. Although final results are not yet available, these results are encouraging.

### Modified Conventional Chemotherapeutics

PEP02 (MM-398, Merrimack Pharmaceuticals) is a novel nanoparticle liposomal formulation of irinotecan that has improved pharmacokinetics and tumor biodistribution. In a recent Phase II clinical trial of 40 patients with metastatic gemcitabine-refractory PDA, PEP02 120 mg/m^2^ every 3 weeks showed a median PFS and OS of 9 and 21.6 weeks, respectively. Grade 3/4 adverse events included cytopenias, diarrhea and fatigue [55]. An international Phase III clinical trial (NAPOLI 1; NCT01494506) of this product in combination with 5-FU is underway.

S-1(Teysuno®) is a fourth generation oral fluoropyrimidine that contains tegafur, gimeracil, and oteracil potassium. In a Japanese phase II, randomized trial (PC-01 Study), 116 patients with stages III–IV PDA were randomized to gemcitabine plus S1 or placebo. Median (13.7 vs. 8.0 months) and 1-year (29 % vs. 55.9 %) OS was better in the S-1 group (*P* = 0.035). However, treatment discontinuation due to toxicity was seen in 27 % of the S-1 group [56]. S-1 is currently approved for treatment of gastrointestinal malignancies in Asia and Europe. A Phase III trial (JASPAC-01) of 385 patients comparing S-1 to gemcitabine for adjuvant therapy of resected PDA. In this study powered for non-inferiority, S-1 was not only non-inferior (*P* < 0.001) but also superior (*P* < 0.001) to gemcitabine. The authors concluded that S-1 was better than gemcitabine and should be considered the new standard for resected PDA [57].

High-dose intravenous (IV) vitamin C (ascorbate) has been shown to synergize with gemcitabine leading to enhanced killing of tumor cells by a pro-oxidative mechanism. Two Phase I studies have shown that combination of pharmacological doses of ascorbate with gemcitabine are safe in advanced pancreatic cancer [58, 59]. In the first, 8 of 14 patients treated with gemcitabine and erlotinib in combination with high-dose IV vitamin C had a reduction in the size of the pancreatic primary. In the second, among nine patients PFS was 26 weeks and OS was 12 months. Two Phase II trials are currently ongoing testing the efficacy of high-dose IV ascorbate to enhance efficacy of gemcitabine-based chemotherapy.

## Key Negative Trials

We will briefly discuss key Phases II–III clinical trials that were negative, in spite of a strong biological rationale and/or promising Phase II results. Table 2 serves as a summary.

**Table 2:** Summary of key negative clinical trials in metastatic PDA

Intervention	Study description	No. of patients	Comparison	Median OS	Median DFS or TTP	ORR	1-year survival	References
*Bevacizumab*								
Phase 2	Single arm; multicenter, single institution	52	None	8.8 (7.4–9.7 months)	5.4 (3.7–6.2 months)	21 %	29 %	Kindler et al. [39]
Phase 3	Randomized, double-blind, placebo-controlled, international	602	Gemcitabine ± bevacizumab or Placebo	5.9 vs. 5.8 months	3.8 vs. 2.9 months	10 % vs. 13 %	Not reported (~15 % both)	Kindler et al. [60]
*Cetuximab*								
Phase 2	Single arm; multicenter	41	None	7.1 months	3.8 months	12 %	31.7 %	Xiong et al. [65]
Phase 3	Randomized, double-blind, placebo-controlled	745	Gemcitabine ± Cetuximab or Placebo	6.3 vs. 5.9 months	3.4 vs. 3.0 months	12 % vs. 14 %	Not reported (~20 % both)	Philip et al. (2010) [62]
*Ganitumab*								
Phase 2	Randomized, Placebo-controlled	125	Gemcitabine ± Ganitumab, Conatumumab or Placebo	8.7 vs. 7.5 vs. 5.9 months	5.1 vs. 4.0 vs. 2.1 months	10 % vs. 3 % vs. 3 %	39 % vs. 20 % vs. 23 %	Kindler H, et al. (2012)[69]
*Axitinib*								
Phase 2	Randomized, multi-center, open label	103	Gemcitabine ± Axitinib or Placebo	6.9 vs. 5.6 months	4.2 vs. 3.7 months	7 %	37 % vs. 23.5 %	Spano et al. [62]
Phase 3	Randomized, double-blinded	632	Gemcitabine ± Axitinib or Placebo	8.5 vs. 8.3 months	4.4 months (both)	5 % vs. 2 %	Not reported	Kindler et al. (2011) [61]

### Anti-VEGF

In 2010, the CALGB reported the results of a randomized Phase III clinical trial in which 602 patients with stages III–IV PDA were randomized to receive either gemcitabine alone or in combination with bevacizumab [39]. The results were disappointing with no difference in median OS (5.8 vs. 5.9 months). In the preceding single-arm Phase II study, a median 8.8 month OS and 21 % ORR was seen in 52 patients with metastatic disease [60]. In reflection, the results of this small, single-arm study were not significantly better than what was previously published for gemcitabine alone. Fixed dose rate gemcitabine plus bevacizumab followed by chemo-radiation in LA potentially resectable disease fared no better [61].

Axitinib, an oral and selective inhibitor of VEGFs, currently approved in the USA for advanced renal cell cancer, was also tested in PDA. In a randomized placebo-controlled Phase II study, 103 patients with unresectable or metastatic PDA were treated with gemcitabine, with or without axitinib. The ORR for gemcitabine plus axitinib was 7 % vs. 3 % for gemcitabine alone, and the median OS was 6.9 vs. 5.6 months, respectively [62]. Investigators interpreted this non-statistically significant prolongation of 1.3 months as a signal of activity and proceeded with a Phase III clinical trial enrolling 632 patients [63]. In a pre-planned interim analysis done in January 2009, an independent committee concluded the study was futile and it was terminated. The reported median OS was 8.5 vs. 8.3 months (HR = 1 · 014; 95 % CI: 0 · 786–1 · 309).

### Cetuximab

At the same time that CALGB reported its negative study with bevacizumab, the SWOG group released the results of a randomized Phase III clinical trial of similar design using cetuximab instead of bevacizumab. The median OS was 6.3 vs. 5.9 months for the experimental [64]. Again, the preceding study was a single-arm Phase II study of the combination of cetuximab and gemcitabine that included only 41 patients. Median OS of 7.1 months and a 12 % RR was felt to be encouraging, but not significantly higher than gemcitabine alone [65]. Negative results for this combination were also observed in the adjuvant setting [66].

### Insulin-Like Growth Factor Receptor (IGF-R) Blockade

Blockade of IGF-R1 is underpinned by strong pre-clinical data [67, 68]. Ganitumab, Amgen's human monoclonal antibody that targets IGF-1R, was studied in combination with gemcitabine in a Phase II trial of 42 patients with metastatic disease. Median OS of 8.7 months and 1-year survival rates were 39 % [69], slightly better than placebo. In August 2012, following a preplanned interim analysis of the Phase III GAMMA trial, Amgen stopped the study because the addition of ganitumab to gemcitabine was unlikely to demonstrate improvements in OS. Similarly, anti-IGF-1R monoclonal antibody cixutumumab (ImClone) in combination with gemcitabine/erlotinib did not improve PFS or OS in metastatic PDA [70]. A Phase II three-arm study of dalotuzumab (MK-0646, Merck) in combination with gemcitabine with or without erlotinib (NCT00769483) was recently completed.

### Gemcitabine Elaidate (CO-1.01, CP-4126)

Gemcitabine elaidate is a lipophilic, unsaturated fatty acid ester derivative of gemcitabine (Clovis) designed to allow the drug to enter tumoral cells by passive diffusion and therefore independently of the human equilibrative nucleoside transporter-1 (hENT1). The pivotal LEAP study, a randomized Phase II international clinical trial, required the measurement of hENT in metastatic biopsies prior to randomization, hoping to correlate hENT expression with gemcitabine benefit. A number of 361 patients were enrolled rapidly, but unfortunately, no difference in OS was found in either the hENT1-low or in the overall intent-to-treat population. The study also demonstrated that hENT1 status has no clinical utility for determining gemcitabine sensitivity [71].

## Future Directions in the Adjuvant and Neoadjuvant Settings

At the current time, we do not know the benefit of multi-agent chemotherapy in the adjuvant setting and therefore we still rely on results of CONKO-1 and ESPAC-3 that demonstrated a role for single-agent gemcitabine and 5-FU. The testing of combination chemotherapy regimens in resectable and LA disease is ongoing and hopefully will also lead to clinical advances. Table 3 summarizes some of the most relevant ongoing clinical trials.

**Table 3:** Selected active phase II and III ongoing adjuvant and neo-adjuvant clinical trials

Mechanism of action/sponsor	Clinical setting/Identifier	Phase	Contol arm	Population	Number targeted	Primary outcome	Comments
*Conventional chemotherapy*							
mFOLFIRINOX (UNICANCER)	Adjuvant (NCT01526135)	III	Gem	Resected (R0-R1)	490	DFS	Arm A: Gem
							Arm B: mFOLFORINOX (No 5-FU bolus)
mFOLFIRINOX (Stanford University)	Neo-adjuvant (NCT01926197)	III	mFOLFIRINOX	Locally advanced, UR	172	PFS	Arm A: mFOLFORINOX
							Arm B: mFOLFORINOX → 5-FU CR (SBRT)
FOLFIRINFOX (Univer. Erlangen-Nürnberg)	Neo-adjuvant (NCT01827553)	III	Gem	Locally advanced UR	830	OS	Arm A: induction FOLFIRINOX → Gem CR or FOLFIRINOX
							Arm B: induction Gem → Gem CR or Gem
Gemcitabine + Oxaliplatin (University Zurich)	Neo-adjuvant (NCT01314027)	III	Gem	Resectable	310	PFS	Arm A: Neoadj GemOx → Surgery → Adj Gem
							Arm B: Surgery → Adj Gem
Gemcitabine + NabPaclitaxel (University Florida)	Neo-adjuvant (NCT01470417)	II	None	Resectable & BR	60	RR	High-risk resectable/BR disease receive GemNab-pac → GEM CR
*Immunotherapy*							
Algenpantucel-L (NewLink Genetics)	Adjuvant (NCT01072981)	III (completed)	Gem ± CR	Resected (R0–R1)	722	OS	Arm A: Surgery → Gem ± 5FU CR
							Arm B: Surgery → Algenpantucel-L + Gem ± CR
Algenpantucel-L (NewLink Genetics)	Neo-adjuvant (NCT01836432)	III	FOLFIRINOX	BR and locally advanced UR	280	OS	Arm A: FOLFIRINOX → 5FU CR
							Arm B: Algenpantucel-L + FOLFIRINOX → 5FU CR
*Targeted agents*							
Erlotinib (RTOG)	Adjuvant (NCT01013649)	III	Gem	Resected, R0-R1	950	OS	Double randomization: Gem vs. Gem + erlotinib → 5FU CR vs. no 5FU CR
Erlotinib (ACOS)	Neo- adjuvant (NCT00733746)	II	None	Potentially resectable	123	2-year OS	Gem + erlotinib pre- and post-surgery

*Gem* gemcitabine, *m* modified, *Adj* adjuvant, *CR* chemoradiation (unless otherwise specified = 50.4 cGy), *BR* borderline resectable, *UR* unresectable

The majority of the larger active trials are testing conventional chemotherapy agent combinations. In the adjuvant setting, the first trial that results are expected to be available is the recently completed NewLink Genetics Phase III study that compares standard of care gemcitabine with and without HyperAcute-Pancreas immunotherapy (Algenpantucel-L) followed by chemo-radiation. FOLFIRINIOX is being formally tested in surgically resected disease by French investigators (UNICANCER Group) in a Phase III clinical trial in which subjects with R0 or R1 resections are randomized to a modified version of FOLFIRINOX or standard gemcitabine. The target number is 490 and the estimated completion date is in 2018. The Phase III NCI-sponsored trial RTOG-0848 compares erlotinib in combination with gemcitabine vs. gemcitabine alone in the first randomization and the additional benefit of chemoradiation in the second randomization. Estimated enrollment is 950, with estimated completion of the primary outcome measure (OS from 1st randomization) targeted for 2020. Enrollment has been slow to this trial, necessitating the addition of additional cooperative groups and sites worldwide. An interesting Phase II ACOS trial centered at MD Anderson also assesses the benefit of gemcitabine and erlotinib in surgically-resectable disease. In this study, two cycles of the 3 weeks on 1-week off combination is given prior to resection and reinitiated post-pancreatiduodenectomy. Blood and tissue analysis is planned for correlative studies. An alternative adjuvant approach is the two-arm Phase III trial from Germany comparing gemcitabine alone to gemcitabine plus cisplatin with regional hyperthermia (HEAT, NCT01077427). The preceding Phase II trial demonstrated a low toxicity rate and an OS of 16.9 months.

FOLFIRINOX has already been shown to be an effective treatment option in LA PDA as demonstrated by several single institution studies, with response rates as high as 50 % [14–16]. German investigators will assess the effectiveness of chemotherapy and chemoradiation vs. chemotherapy alone in LA unresectable pancreatic cancer in the four-arm CONKO-7 trial. A total of 830 patients will first receive either induction FOLFIRINOX or gemcitabine. Following completion of induction chemotherapy subjects will then receive 50.4 Gy radiation plus radiosensitizing gemcitabine OR continue with the same chemotherapy. The expected completion date of this trial is 2018. A Phase II trial of similar design is being conducted at MD Anderson (NCT01560949). Investigators at Stanford University also plan to test FOLFIRINOX in combination with chemoradiation; however, in this trial, subjects will undergo treatment with modified FOLFIRINOX plus or minus SBRT radiation with 5-FU as the radiosensitizing agent. Secondary objectives include evaluation of the utility of FDG- PET and biomarker identification.

The University of Florida has launched the Phase II GAIN-1 trial in resectable and borderline-resectable pancreatic cancers testing gemcitabine + nab-paclitaxel as neoadjuvant therapy. Serological (CA 19.9), radiological, and pathological response rate are the main outcomes. Lastly, European researchers are currently testing another commonly used regimen (gemcitabine + oxaliplatin) in a Phase III clinical trial. Patients assigned to the control arm will receive standard surgical resection followed by adjuvant gemcitabine. Patients on the investigational arm will receive 8 weeks of neoadjuvant gemcitabine/oxaliplatin chemotherapy followed by surgery in addition to gemcitabine adjuvantly. Preliminary results of this trial will be available by the end of 2014.

## Conclusions

We have summarized here the most recent treatment advances in PDA, including the results of the FOLFIRINOX and nab-paclitaxel plus gemcitabine Phase III trials. We discussed novel approaches such as immune-mediated therapies, drugs designed to disrupt the tumor-stromal compartment and to take advantage of genetic defects in DNA repair as well as new generations of conventional chemotherapeutics, all in early stages of clinical development with promising results. We hope that at least some of these agents will result in positive trials and add to our armamentarium for treating this challenging malignancy.

We are encouraged by recent leaps forward in the treatment of PDA. Future research will focus on identifying molecularly based clinical subtypes of pancreatic cancer and the further development of criteria to assist in our clinical decision-making. The long sought after goal of personalizing drug choices based on pancreatic tumor and patient biology will hopefully be possible. Can we define better biomarkers particularly for guidance in the neoadjuvant treatment of potentially resectable disease? How will these recent advances, primarily in the treatment of metastatic disease, help to improve outcomes for surgically-amenable pancreatic cancer? This of course is the treatment goal we are most asked by our patients cure? With the availability of effective treatments for metastatic PDA, we are now able to work toward this goal.
